# The optimum titanium precursor of fabricating TiO_2_ compact layer for perovskite solar cells

**DOI:** 10.1186/s11671-017-2418-9

**Published:** 2017-12-29

**Authors:** Jianqiang Qin, Zhenlong Zhang, Wenjia Shi, Yuefeng Liu, Huiping Gao, Yanli Mao

**Affiliations:** 1 School of Physics and Electronics, Henan University, Kaifeng, 475004 China; 2 Institute of Micro/Nano Photonic Materials and Applications, Henan University, Kaifeng, 475004 China

**Keywords:** Perovskite solar cells, Compact layer, Titanium precursor

## Abstract

Perovskite solar cells (PSCs) have attracted tremendous attentions due to its high performance and rapid efficiency promotion. Compact layer plays a crucial role in transferring electrons and blocking charge recombination between the perovskite layer and fluorine-doped tin oxide (FTO) in PSCs. In this study, compact TiO_2_ layers were synthesized by spin-coating method with three different titanium precursors, titanium diisopropoxide bis (acetylacetonate) (c-TTDB), titanium isopropoxide (c-TTIP), and tetrabutyl titanate (c-TBOT), respectively. Compared with the PSCs based on the widely used c-TTDB and c-TTIP, the device based on c-TBOT has significantly enhanced performance, including open-circuit voltage, short-circuit current density, fill factor, and hysteresis. The significant enhancement is ascribed to its excellent morphology, high conductivity and optical properties, fast charge transfer, and large recombination resistance. Thus, a power conversion efficiency (PCE) of 17.03% has been achieved for the solar cells based on c-TBOT.

**Electronic supplementary material:**

The online version of this article (10.1186/s11671-017-2418-9) contains supplementary material, which is available to authorized users.

## Background

In 2009, hybrid organic-inorganic perovskite material MAPbI_3_ was firstly reported on solid-state solar cells as light absorber [1]. Perovskite solar cells (PSCs) have attracted tremendous attentions due to its high performance and rapid efficiency promotion [2]. Over the past 5 years, the power conversion efficiency (PCE) of PSCs has rapidly increased from 9 to 22.1% [3]. In general, PSCs are made up of compact layer, electron transfer layer, perovskite absorber layer, and hole transfer layer (HTL). Subsequently, some new structures were fabricated, such as planar PSCs (without mesoporous TiO_2_ (mp-TiO_2_) layer) [4, 5] and PSCs without HTL [6]. However, it is widely recognized that compact TiO_2_ (c-TiO_2_) layer is always an indispensable part for high-performance PSCs. On the one hand, it can act as the electron transport layer to transport electrons generated from perovskite layer [7]. On the other hand, it can serve as the block layer to hinder direct contact between the holes and FTO [7, 8].

Currently, various methods of fabricating c-TiO_2_ have been put forward in early literature, such as spray pyrolysis [9], spin-coating [10], atomic layer deposition (ALD) [11], sputtering [12], and electrochemical deposition [13]. Especially, spin-coating is widely used in PSCs due to its low-cost, simplicity, and convenience. According to early reports, the titanium precursor solutions were commonly prepared by using titanium diisopropoxide bis (acetylacetonate) (c-TTDB) [14] and titanium isopropoxide (c-TTIP) [15] as titanium sources. Du et al. [16] reported the c-TiO_2_ layer prepared by using tetrabutyl titanate (c-TBOT) as titanium source. Until today, the optimization of compact layer also attracts much attention. Tu et al. [17] provided a low-cost and efficient method to fabricate compact layer by TiO_2_ quantum dots. Tan et al. [18] reported a simple method using Cl-TiO_2_ as compact layer at low temperature (< 150 °C), which exhibit a high PCE and stability. However, there are few studies on which titanium precursor is more suitable for c-TiO_2_ prepared by spin-coating method in PSCs.

In this work, we have synthesized the c-TiO_2_ by three kinds of titanium precursor solutions with different titanium sources, i.e., c-TBOT, c-TTIP, and c-TTDB. Subsequently, the properties of the c-TiO_2_ and their effects on the performance of PSCs have been systematically investigated. Compared with the widely used c-TTDB and c-TTIP, c-TBOT is the better choice due to its high conductivity, transmittance, charge extraction capacity, and low carrier recombination. Accordingly, the PSCs based on c-TBOT show higher open-circuit voltage (*V*_oc_), short-circuit current density (*J*_sc_), fill factor (FF), and lower hysteresis, yielding a higher PCE. An average PCE of 17.03% was obtained from the cells based on c-TBOT.

## Experimental

### Preparation of compact TiO_2_ layers

Firstly, FTO glass substrates (~ 15 Ω/Sq, Japan) were etched by 2 M HCl and Zn powder. Secondly, the substrates were cleaned in Hellmanex detergent, deionized water, acetone, 2-propanol, and ethanol, respectively. Last, the substrates were treated by UV-O_3_ for 15 min. The compact layer was deposited on FTO glass by spin-coating at 3000 rpm for 30 s and annealed at 500 °C for 30 min.

Three different titanium precursor solutions were prepared as follows. The precursor solution for c-TBOT: 0.25 mL tetrabutyl titanate (99%, Aladdin reagent) was diluted in 5 mL ethanol, followed by adding 0.2 g polyethylene glycol, 1 mL nitric acid, and 0.5 mL deionized water. Then, the mixed solution was stirred for 5 h and precipitated for 15 h. Last, the mixture was filtered with 0.45 μm PTFE filter [16]. As for c-TTDB, the precursor solution consists of 0.15 M titanium diisopropoxide bis (acetylacetonate) (75 wt% in isopropanol, Sigma-Aldrich) in 1-butanol [14]. As for c-TTIP, the precursor solution is composed of 0.23 M titanium isopropoxide (99.999%, Aladdin reagent) and 0.013 M HCl in isopropanol. Firstly, 369 μL titanium isopropoxide and 35 μL 2 M HCl solutions were diluted in 2.53 mL isopropanol, separately. Next, the HCl solution was added in titanium precursor drop by drop under heavy stirring. Last, the mixture was filtered with 0.45 μm PTFE filter [19].

### Device fabrication

The mp-TiO_2_ layer was coated on the c-TiO_2_ layer by spin-coating TiO_2_ paste diluted in ethanol (the weight ratio 1:6) at a speed of 4000 rpm for 30 s, followed by heating at 100 °C for 10 min and annealing at 500 °C for 30 min, respectively. Then, the perovskite layer was deposited on the mp-TiO_2_ by anti-solvent method previously reported [9]. In brief, the precursor was prepared in a glovebox containing FAI (1 M), PbI_2_ (1.1 M), MABr (0.2 M), and PbBr_2_ (0.2 M) in a mixed solution of DMF and DMSO (the volume ratio 4:1). The solution was deposited on the mp-TiO_2_ layer by spin-coating in two steps at 1000 rpm for 10 s and 4000 rpm for 30 s. Two hundred microliters of chlorobenzene was dropped on the substrate during the second step before the end of 20 s. Then, the substrates were heated on the hotplate at 100 °C for 1 h. Subsequently, the spiro-OMeTAD solution was coated on the perovskite layer by spin-coating at speed of 4000 rpm for 30 s after the substrates cooled down to room temperature. The spiro-OMeTAD solution consists of 72.3 mg spiro-MeOTAD, 28.8 μL TBP (4-tert-butylpyridine), 17.5 μL lithium bis (trifluoromethanesulfonyl) imide (Li-TFSI) solution (520 mg Li-TFSI in 1 mL acetonitrile), and 1 mL chlorobenzene. Finally, a 70-nm-thick gold electrode was deposited on the top of HTL by thermal evaporation.

### Characterization

The morphology and microstructure of the compact layer were observed by field emission scanning electron microscope (FESEM, JEM-7001F, JEOL) and scanning probe microscope (Multimode 8, Bruker, America). X-ray diffraction (XRD) patterns were characterized by a diffractometer (D8 Advance, Bruker, Germany) with Cu-Kα source (*λ* = 0.1542 nm). Current density-voltage (*J*-*V*) curves of the devices were performed by using a source meter (Keithley 2440) and under standard illumination (AM 1.5 G, 100 mW cm^−2^) from a Newport Oriel Solar Simulator. The active area of the solar cells is 0.1 cm^2^ defined by a shadow mask. Conductivity measurements of TiO_2_ films were measured by using a source meter (Keithley 2400). Steady-state photoluminescence and time-resolved photoluminescence were measured by FLS 980E fluorometer (Edinburgh Photonics). The UV-vis absorption spectra were conducted using a UV-vis spectrophotometer (Cary 5000 UV-vis-NIR). Electrochemical impedance spectroscopy measurement was carried out by an electrochemical workstation (CHI660e, Shanghai CHI Co., Ltd) with forward biases of 0.8 V in the frequency range of 0.1 Hz to 1 MHz under AM1.5G. The amplitude of the signal was 10 mV. The incident photon-to-current conversion efficiency (IPCE) was recorded by a solar cell IPCE measurement system (Crowntech Qtest Station 500ADX, America).

## Results and discussion

**Fig. 1:**
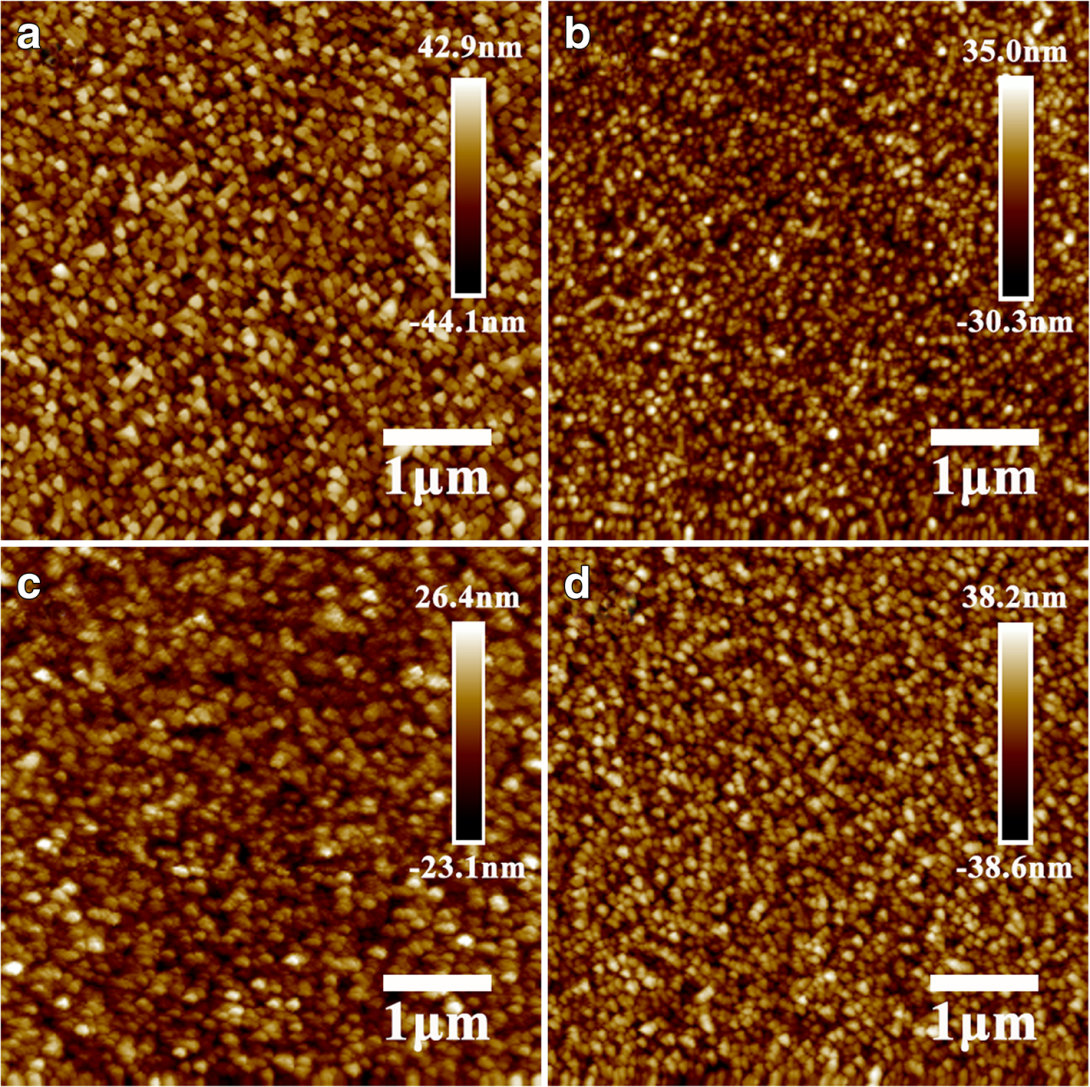
AFM images of **a** bare FTO, **b** c-TBOT, **c** c-TTIP, and **d** c-TTDB

Figure 1a–d shows the atomic force microscope (AFM) images of compact layers. Compared with c-TBOT and c-TTDB, the sample of c-TTIP exhibits a relatively smoother surface. In addition, the root mean square (RMS) roughness values of the various substrates on a 5 μm × 5 μm scale are listed in Additional file 1: Table S1. The RMS roughness value of the FTO is 13.4 nm, which gradually decrease to 11.4, 9.38, and 6.65 nm after coated with c-TTDB, c-TBOT, and c-TTIP, respectively. After coated with c-TiO_2_, the substrates become much more uniform and smoother. It suggests that the TiO_2_ layers have been successfully coated on the FTO.

In order to investigate the morphology and thickness of the compact layers, scanning electron microscope (SEM) measurements were performed. Additional file 1: Figure S1a–g shows the top-view and cross-sectional SEM images of different c-TiO_2_ layers. The compact layers prepared by different precursors reveal different surface morphology. The thickness of c-TTDB is slightly thinner (35 nm) than that of c-TTIP (50 nm) or c-TBOT (45 nm), which can be attributed to the different adhesion of precursor solutions. In addition, cyclic voltammetry (CV) is a sensitive method to detect the pinhole defects of the compact layers [20]. The CV measurements of the compact layer formed with different precursor solutions were performed, and the results were shown in Additional file 1: Figure S2. Compared with c-TTDB and c-TTIP, c-TBOT reveals fewer pinhole defects and better blocking function.

**Fig. 2:**
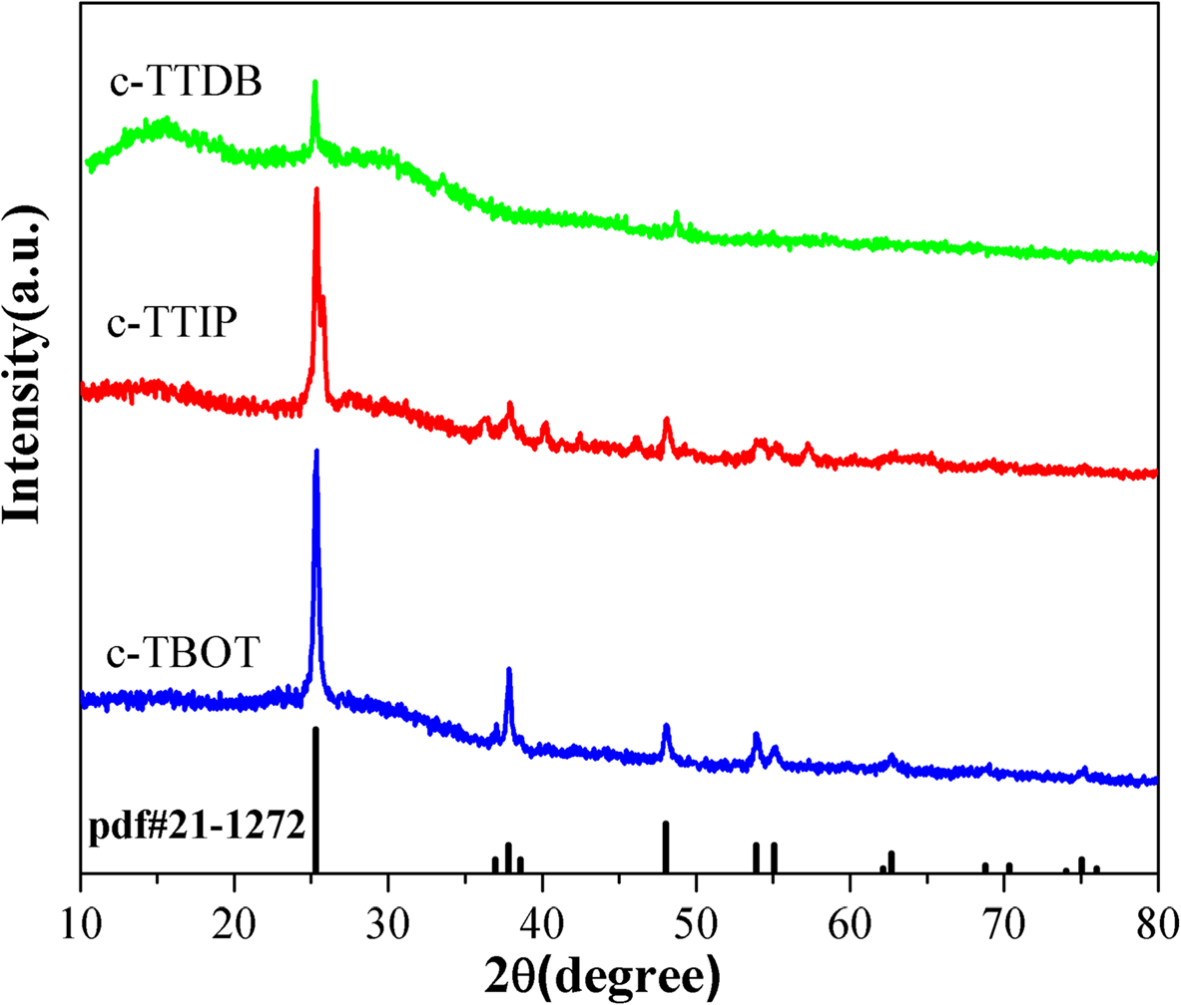
X-ray diffraction patterns of c-TBOT, c-TTIP, and c-TTDB deposited on glass without FTO

Figure 2 shows the XRD patterns of c-TiO_2_ deposited on the glass without FTO by multilayer coating. The c-TTDB shows a weak peak at 2θ = 25.3°, which correspond to the (101) plane of anatase phase (JCPDS card no. 21-1272). Similarly, c-TTIP exhibits an obvious anatase peak at 2θ = 25.3°. This result is consistent with the previous report in literature [21, 22]. As for c-TBOT, the diffraction peaks at 2θ = 25.3°, 37.8°, 48.0°, and 53.8° are assigned to the anatase planes of (101), (004), (200), and (105), respectively. Compared with c-TTIP and c-TTDB, c-TBOT shows the larger intensity and narrower full width at half maximum (FWHM) anatase diffraction peaks, which may be ascribed to the different thickness and crystallinity of the films [23].

**Fig. 3:**
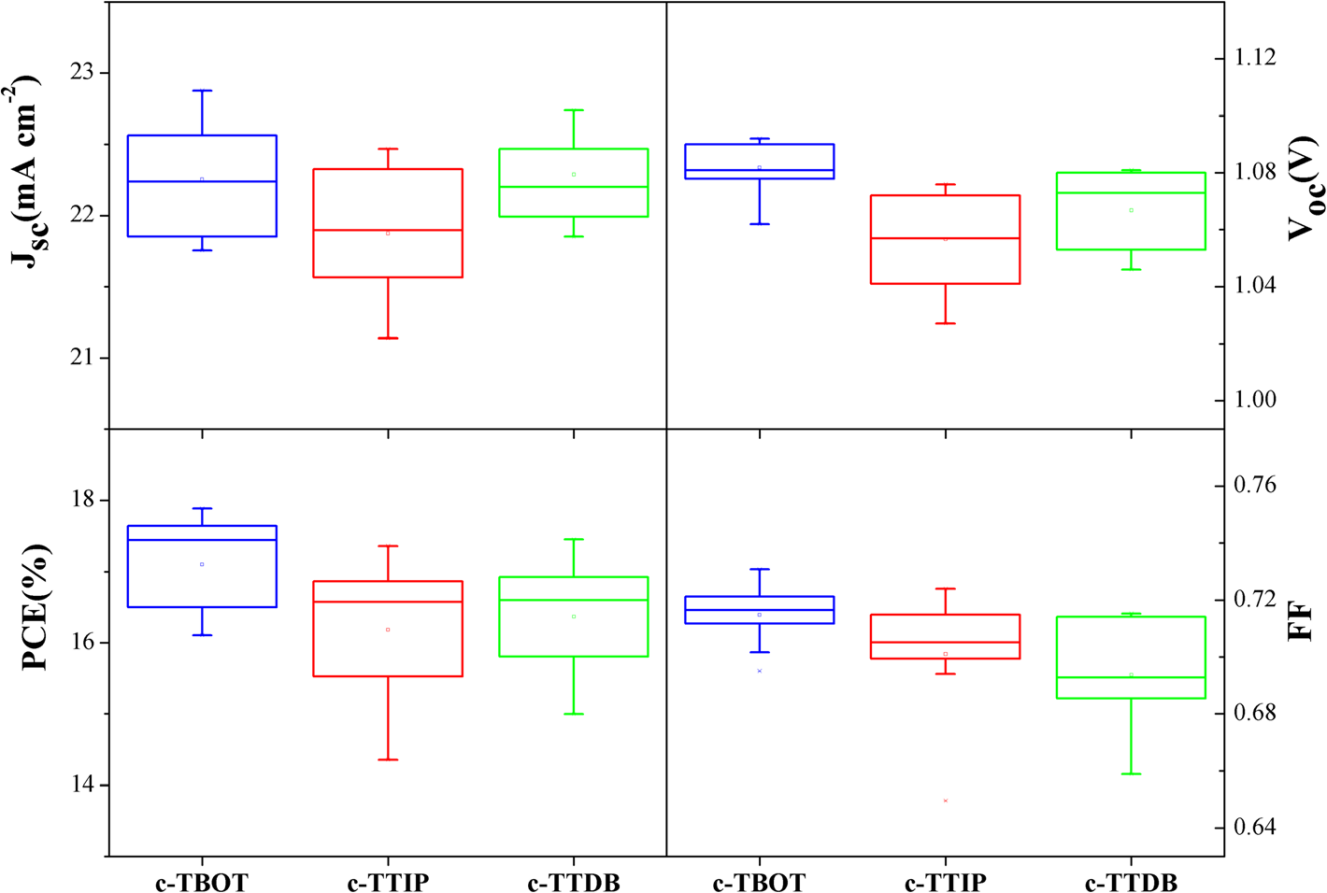
Photovoltaic parameters of devices plotted as a function of different compact layers (i.e, *J*_sc_, *V*_oc_, FF, and PCE)

Figure 3 shows the photovoltaic parameters of the devices based on different c-TiO_2_ layers, including *J*_sc_, *V*_oc_, FF, and PCE, respectively. All the photovoltaic parameters were obtained from *J*-*V* curves measured under AM 1.5G and summarized in Table 1. Obviously, the photovoltaic performance is strongly influenced by compact layer. As can be observed, the devices based on c-TBOT show the largest average PCE (17.03%) than those based on c-TTDB (16.22%) and c-TTIP (16.02%). In addition, the other parameters (*J*_sc_, *V*_oc_, FF) of the cells based on c-TBOT are also larger than those based on c-TTDB and c-TTIP. This result indicates that it can improve performance by using c-TBOT as compact layer for PSCs.

**Table 1:** Photovoltaic parameters of the cells with different compact layers

Compact layer	*J*_sc_ (mA/cm^2^)	*V*_oc_ (*V*)	PCE (%)	PCE (max)	FF (%)
c-TBOT	22.19	1.081	17.03	17.88	71.26
c-TTIP	21.83	1.054	16.02	17.32	69.91
c-TTDB	22.18	1.062	16.22	17.60	68.81

**Fig. 4:**
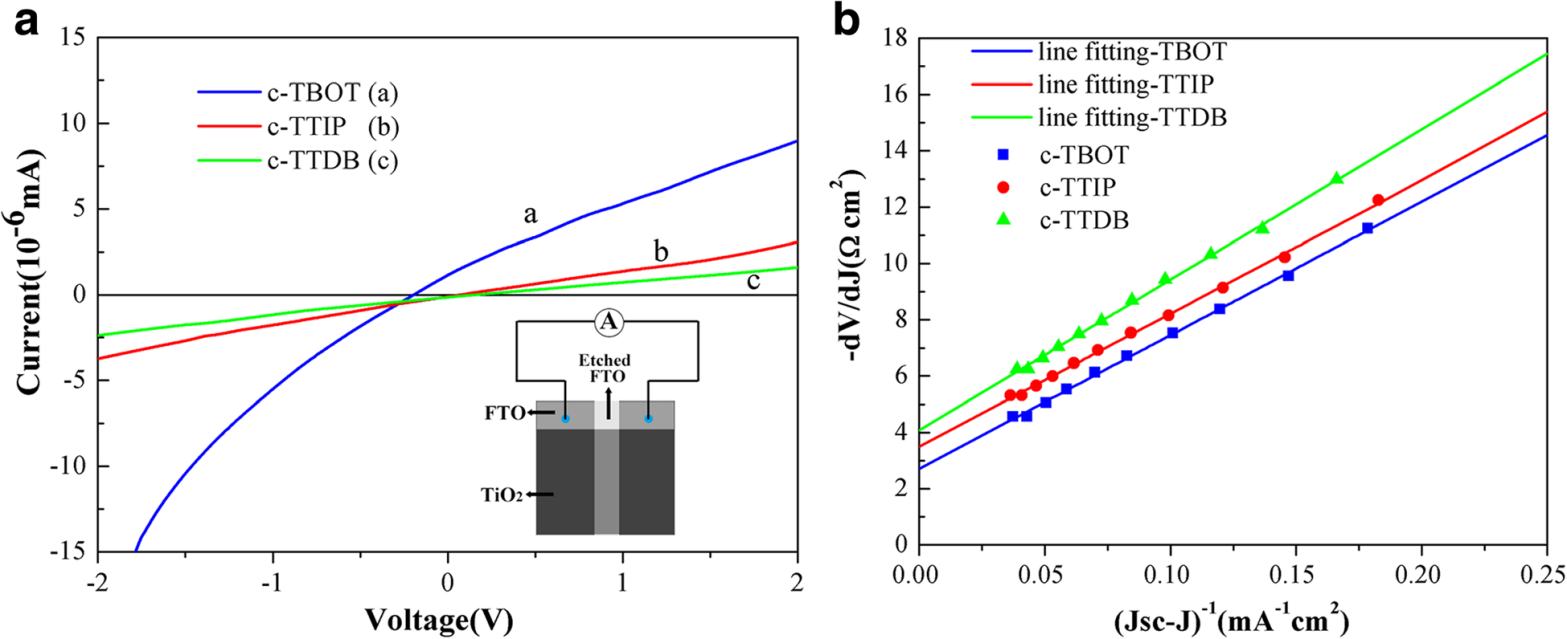
**a** Conductivity measurement results of various c-TiO_2_. The inset depicts the structure of the sample. **b** Plots of − dV/dJ vs (*J*_sc_-*J*)^−1^ derived from *J*-*V* curves and the linear fitting curves

To determine the conductivity of various c-TiO_2_ layers, DC I-V measurements were carried out. The structure for the measurement is shown in the inset of Fig. 4a [24]. As shown in Fig. 4a, the c-TBOT reveals the best conductivity among the samples and the c-TTIP takes the second place.

The series resistance (*R*_s_) of devices fabricated with different compact layers can be calculated from the illuminated *J*-*V* curves. According to previous reports, the *J*-*V* curves of cells can be analyzed with Eq. 1 correlated to an equivalent circuit. Hence, the *R*_s_ can be obtained from Eq. 2 and Fig. 4b [23, 24].

1 $$ J={J}_{\mathrm{L}}-{J}_{\mathrm{o}}\left\{\exp \left[\frac{e\left(V+{\mathrm{JR}}_{\mathrm{s}}\right)}{{\mathrm{AK}}_{\mathrm{B}}T}\right]-1\right\}-\frac{V+{\mathrm{JR}}_{\mathrm{s}}}{R_{\mathrm{s}\mathrm{h}}} $$

2 $$ -\frac{\mathrm{dV}}{\mathrm{dJ}}=\frac{{\mathrm{AK}}_{\mathrm{B}}T}{e}{\left({J}_{\mathrm{s}\mathrm{c}}-J\right)}^{-1}+{R}_{\mathrm{s}} $$

As shown in Fig. 4b, the *R*_s_ of the c-TBOT device (2.71 Ω cm^2^) is smaller than that of c-TTIP (3.50 Ω cm^2^) or c-TTDB (4.08 Ω cm^2^), which is consistent with the resistivity measurement. A lower *R*_s_ is a necessary condition for solar cells with a higher fill factor (FF) [24, 25]. The device based on c-TBOT shows the lowest *R*_s_, so it has the highest FF, which is in good agreement with the results in Table 1.

**Fig. 5:**
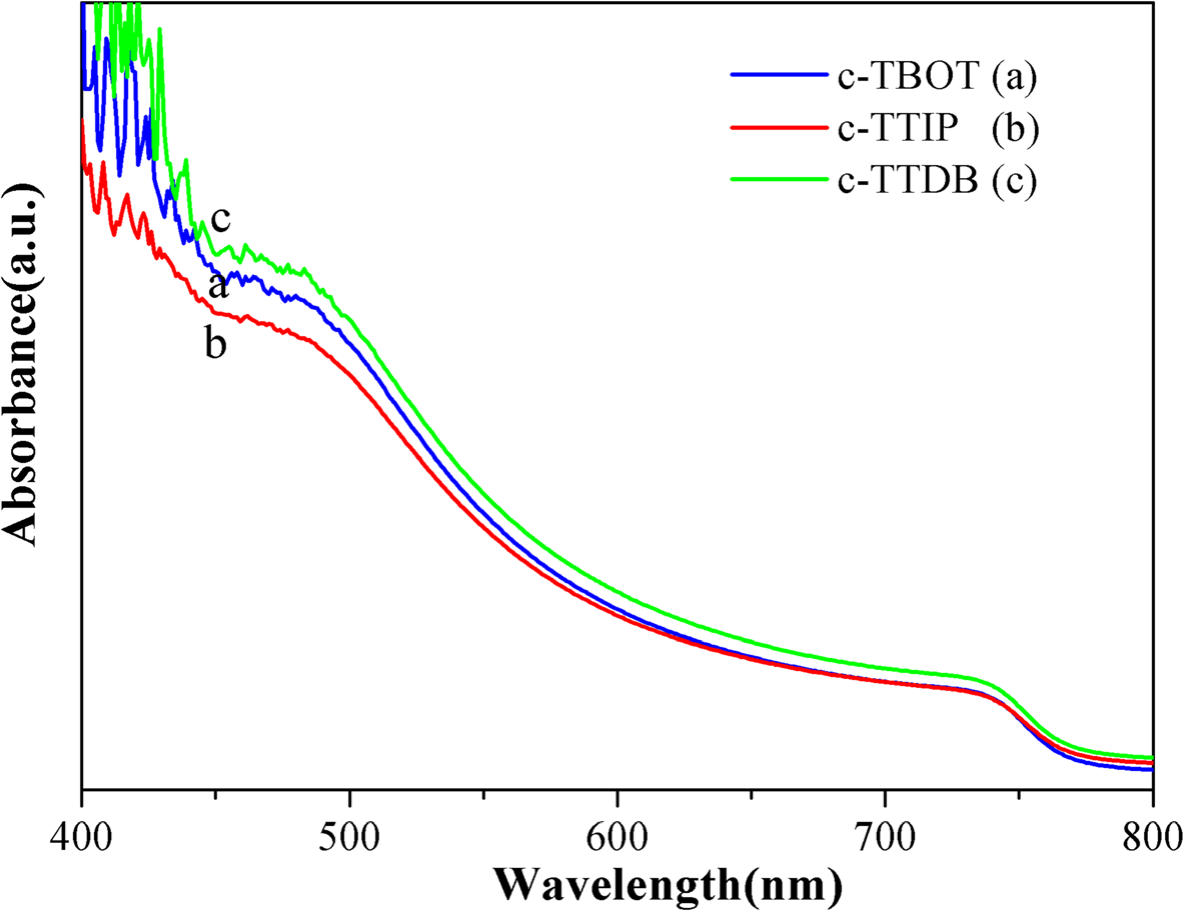
UV-vis absorption spectra of perovskite films based on different compact layers

Figure 5 shows the UV-vis absorption spectra of the perovskite films based on different c-TiO_2_. Obviously, the absorption intensity of the sample based on c-TTDB is the largest and c-TTIP is the weakest in the range of 400–800 nm, which could be attributed to the effect of c-TiO_2_ layers (Additional file 1: Figure S4). Additional file 1: Figure S4 shows the transmission spectra of different c-TiO_2_ layers deposited on FTO glass. All the samples display good light-admitting quality in the range of 350–800 nm. Moreover, the c-TTDB and c-TBOT exhibit higher optical transmission than c-TTIP, which could be ascribed to the different properties of c-TiO_2_ films, such as thickness and roughness. The enhanced light transmission of the c-TiO_2_ certainly increases the light absorption of the perovskite film.

**Fig. 6:**
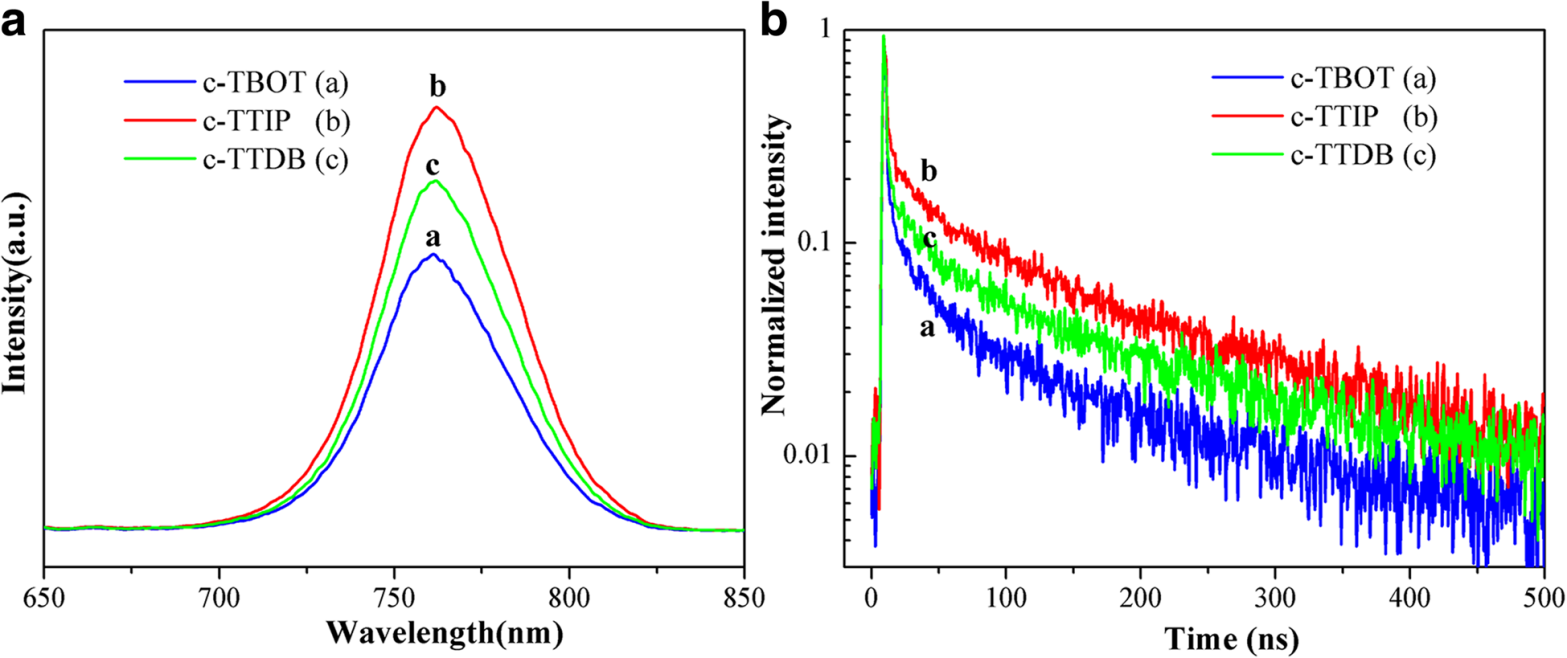
**a** PL and **b** TRPL of perovskite films based on different compact layers

To gain further insight into the charge transfer kinetics between perovskite and TiO_2_, steady-state photoluminescence (PL) and time-resolved photoluminescence (TRPL) were measured. Figure 6a shows the normalized PL spectra of FTO/c-TiO_2_/mp-TiO_2_/perovskite. All the PL spectra exhibit a photoluminescence peak at 770 nm, which is consistent with the early report in literature [9]. The intensity of the PL peaks was decreased in sequence of c-TTIP, c-TTDB, and c-TBOT. The sample of c-TBOT shows the strongest PL quenching due to the faster charge transfer [26, 27]. Meanwhile, Fig. 6b shows the TRPL spectra of FTO/c-TiO_2_/mp-TiO_2_/perovskite. The TRPL curves are fitted a biexponential decay function (Eq. 3), which include a fast decay *τ*_1_ and a slow decay *τ*_2_.

3 $$ I(t)={A}_1\exp \left(\hbox{-} \frac{t}{\tau_1}\right)+{A}_2\exp \left(\hbox{-} \frac{t}{\tau_2}\right) $$

The detailed parameters are summarized in Table 2. The fast decay (*τ*_1_) could be associated with the quenching of free-carrier transfer from perovskite to electron or hole contact. While, the slow decay (*τ*_2_) would be related to the radiative recombination of the charge carries before the charge collection [26, 27]. The perovskite films based on c-TBOT have a slow decay lifetime (*τ*_2_) of 81.39 ns, which is shorter than those based on c-TTDB (97.30 ns) and c-TTIP (109.60 ns). This result indicates that c-TBOT has more efficient charge extraction in cells compared to c-TTDB and c-TTIP [28, 29].

**Table 2:** Parameters of the TRPL spectra

Compact layer	*τ*_1_ (ns)	*τ*_2_ (ns)	Fraction 1 (%)	Fraction 2 (%)
c-TBOT	2.12	81.39	20.15	79.85
c-TTIP	3.05	109.60	9.27	90.73
c-TTDB	2.06	97.30	12.74	87.26

**Fig. 7:**
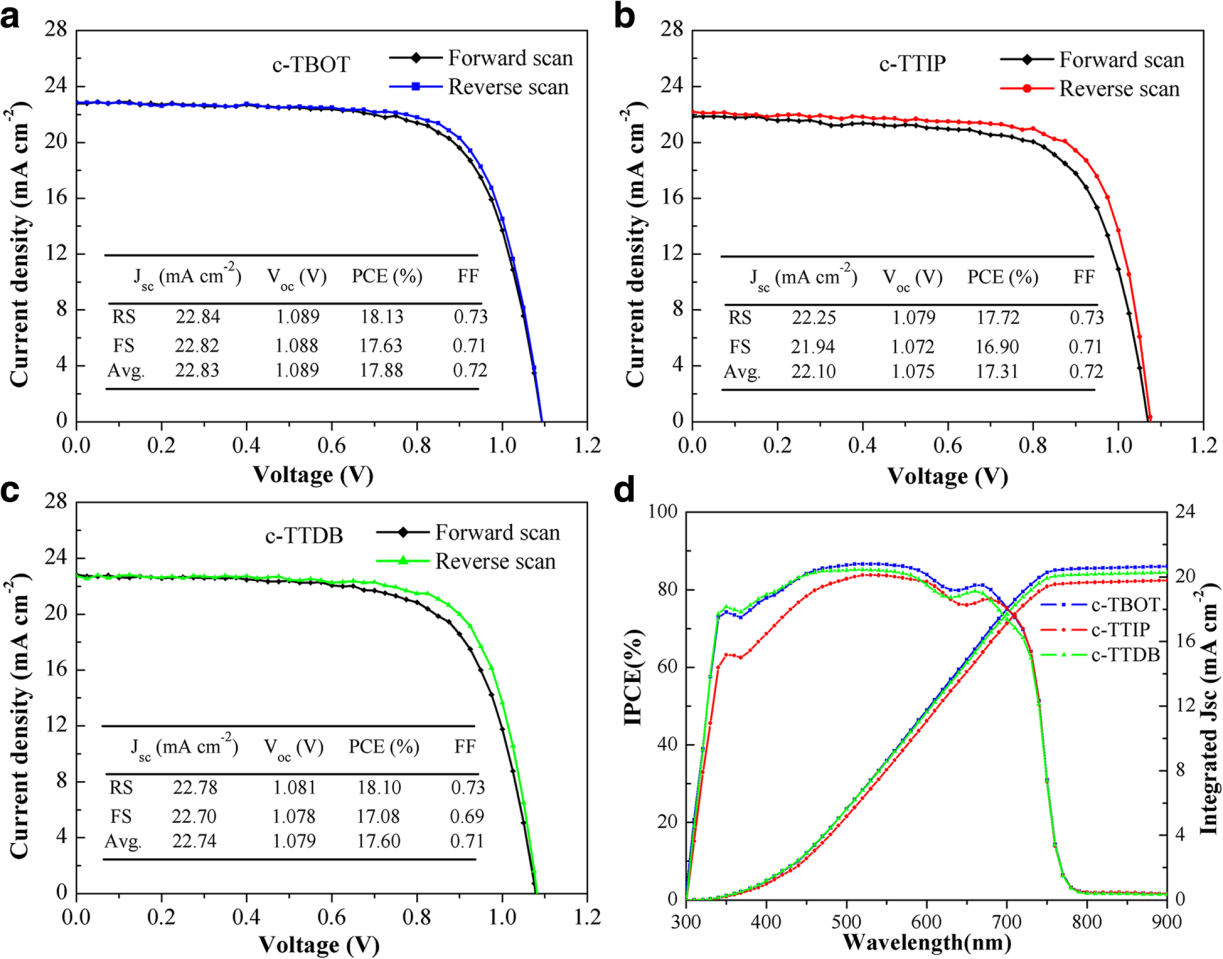
**a**–**d** Current density-voltage (*J*-*V*) curves and IPCE for the best cells based on different compact layers

Figure 7a–c show the *J*-*V* curves of the best performing solar cells fabricated with different compact layers. All the devices based on different compact layers show varying degree of hysteresis between forward and reverse scans. It is generally recognized that the hysteresis is mainly caused by the ion migration, ferroelectric properties of perovskite material, and inadequate charge extraction at interface [30, 31]. Notably, the devices based on c-TBOT reveal a lower hysteresis than those based on c-TTIP and c-TTDB, which is attributed to the superior electron extraction ability at perovskite/TiO_2_ interface [31, 32].

Figure 7d is the incident photon-to-current conversion efficiency (IPCE) spectra of the devices based on different c-TiO_2_ layers. All the IPCE spectra show a broad plateau in the range of 400 to 700 nm. Meanwhile, the IPCE spectra of the devices based on c-TBOT and c-TTDB are higher than that of c-TTIP, which is attributed to the superior light absorption and efficient charge extraction [33, 34], resulting in the higher *J*_sc_. The *J*_sc_ values integrated from IPCE are 20.56, 20.29, and 19.78 mA cm^−2^ for the devices based on c-TBOT, c-TTDB, and c-TTIP, respectively. The integrated *J*_sc_ of the devices based on c-TBOT and c-TTDB are larger than that of c-TTIP, which is in good agreement with the *J*-*V* measurement.

**Fig. 8:**
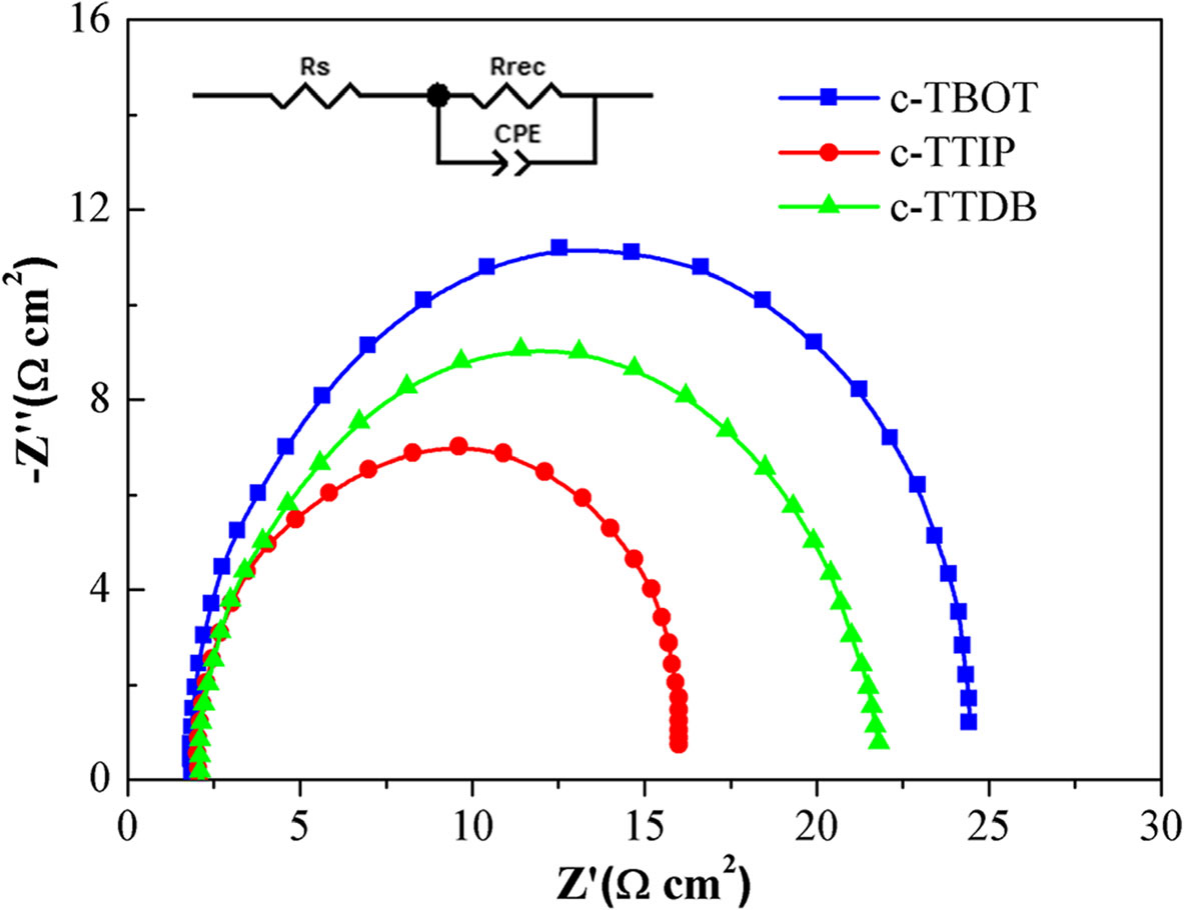
Nyquist plots of the solar cells based on different compact layers at 0.8 V under AM 1.5G. The inset is the equivalent circuit applied to fit the Nyquist plots

To gain further insight into the interfacial charge transport process in PSCs, electrochemical impedance spectroscopy (EIS) measurements were carried out [34]. Figure 8 exhibits the Nyquist plots of the devices based on different c-TiO_2_ layers, and the inset figure depicts the equivalent circuit. According to Nyquist plots, the semicircles observed in mid-frequency region are associated with the charge transfer at the heterojunction interface in PSCs [35]. The fitted parameters for the equivalent circuit are listed in Additional file 1: Table S2. The *R*_s_ value of the cells based on c-TBOT (1.907 Ω cm^2^) is smaller than that of c-TTIP (2.198 Ω cm^2^) or c-TTDB (2.201 Ω cm^2^), which is consistent with the results calculated from the *J*-*V* curves. While, the value of *R*_rec_ based on c-TBOT (22.04 Ω cm^2^) is larger than that of c-TTIP (13.68 Ω cm^2^) or c-TTDB (18.75 Ω cm^2^). The larger *R*_rec_ indicates a lower charge recombination, leading to larger *V*_oc_ [36, 37]. This result agrees well with the *J*-*V* measurement.

## Conclusions

In summary, we have successfully synthesized three kinds of titanium precursor solutions with different titanium sources, i.e., c-TBOT, c-TTIP, and c-TTDB. The photovoltaic parameters of the PSCs based on c-TBOT are higher than those based on c-TTIP and c-TTDB. Additionally, DC I-V measurements show that c-TBOT has high conductivity. The UV-vis absorption spectra exhibit that c-TBOT has excellent optical properties. The PL and TRPL spectra display that the charge transfer for c-TBOT is faster than that for c-TTIP and c-TTDB. The EIS spectra reveal that the charge recombination for c-TBOT is more reduced than the others. All the results can account for the higher *J*_sc_, *V*_oc_, FF, and lower hysteresis. This study proposed a better choice to synthesize high quality compact TiO_2_ layer for PSCs by conventional spin-coating method.

## Additional file

Additional file 1: Figure S1. Top-view SEM images of (a) FTO, (b) c-TBOT, (c) c-TTIP, and (d) c-TTDB. (Each sample of c-TiO_2_ was recoated for 5 times). Cross-sectional SEM images of (e) C-TBOT, (f) c-TTIP, and (g) c-TTDB. (Each sample of c-TiO_2_ was coated for 1 time). **Figure S2.** Cyclic voltammograms at bare FTO and that covered with different c-TiO_2_, scan rate 50 mV s^-1^, electrolyte solution 1 mM K_4_Fe(CN)_6_ + 1 mM K_3_Fe(CN)_6_ in aqueous 0.5 M KCl. **Figure S3.** AFM height profiles of the different c-TiO_2_ spin-coating on the FTO glass obtained from the 5 μm scale, (a) the underlying FTO, (b) c-TBOT, (c) c-TTIP, and (d) c-TTDB. This data can also clearly reveal the roughness of the various substrates. **Figure S4.** Transmission spectra of different compact films deposited on FTO glass. This data was used to analyze the transmissivity of different c-TiO_2_ in the range between 300 and 800 nm. **Table S1.** Parameters of 5 μm × 5 μm AFM roughness. **Table S2.** The fitted parameters for EIS equivalent circuit. (DOC 4192 kb)
