# Supplementary material for: The optimum titanium precursor of fabricating TiO2 compact layer for perovskite solar cells
Source: Nanoscale Res Lett. 2017 Dec 29;12:640. doi: 10.1186/s11671-017-2418-9 (PMC5747562; doi:10.1186/s11671-017-2418-9)
Supplement: Additional file 1: Figure S1. — Top-view SEM images of (a) FTO, (b) c-TBOT, (c) c-TTIP, and (d) c-TTDB. (Each sample of c-TiO2 was recoated for 5 times). Cross-sectional SEM images of (e) C-TBOT, (f) c-TTIP, and (g) c-TTDB. (Each sample of c-TiO2 was coated for 1 time). Figure S2. Cyclic voltammograms at bare FTO and that covered with different c-TiO2, scan rate 50 mV s-1, electrolyte solution 1 mM K4Fe(CN)6 + 1 mM K3Fe(CN)6 in aqueous 0.5 M KCl. Figure S3. AFM height profiles of the different c-TiO2 spin-coating on the FTO glass obtained from the 5 μm scale, (a) the underlying FTO, (b) c-TBOT, (c) c-TTIP, and (d) c-TTDB. This data can also clearly reveal the roughness of the various substrates. Figure S4. Transmission spectra of different compact films deposited on FTO glass. This data was used to analyze the transmissivity of different c-TiO2 in the range between 300 and 800 nm. Table S1. Parameters of 5 μm × 5 μm AFM roughness. Table S2. The fitted parameters for EIS equivalent circuit. (DOC 4192 kb) [file 11671_2017_2418_MOESM1_ESM.doc]

**Supplementary information**

The optimum titanium precursor of fabricating TiO_2_ compact layer for perovskite solar cells

Jianqiang Qin ^a^, Zhenlong Zhang ^a,*^, Wenjia Shi ^a^, Yuefeng Liu ^a^, Huiping Gao ^a^, Yanli Mao ^a,b,*^

^a^ School of Physics and Electronics, Henan University, Kaifeng 475004, China

^b^ Institute of Micro/Nano Photonic Materials and Applications, Henan University, Kaifeng 475004, China


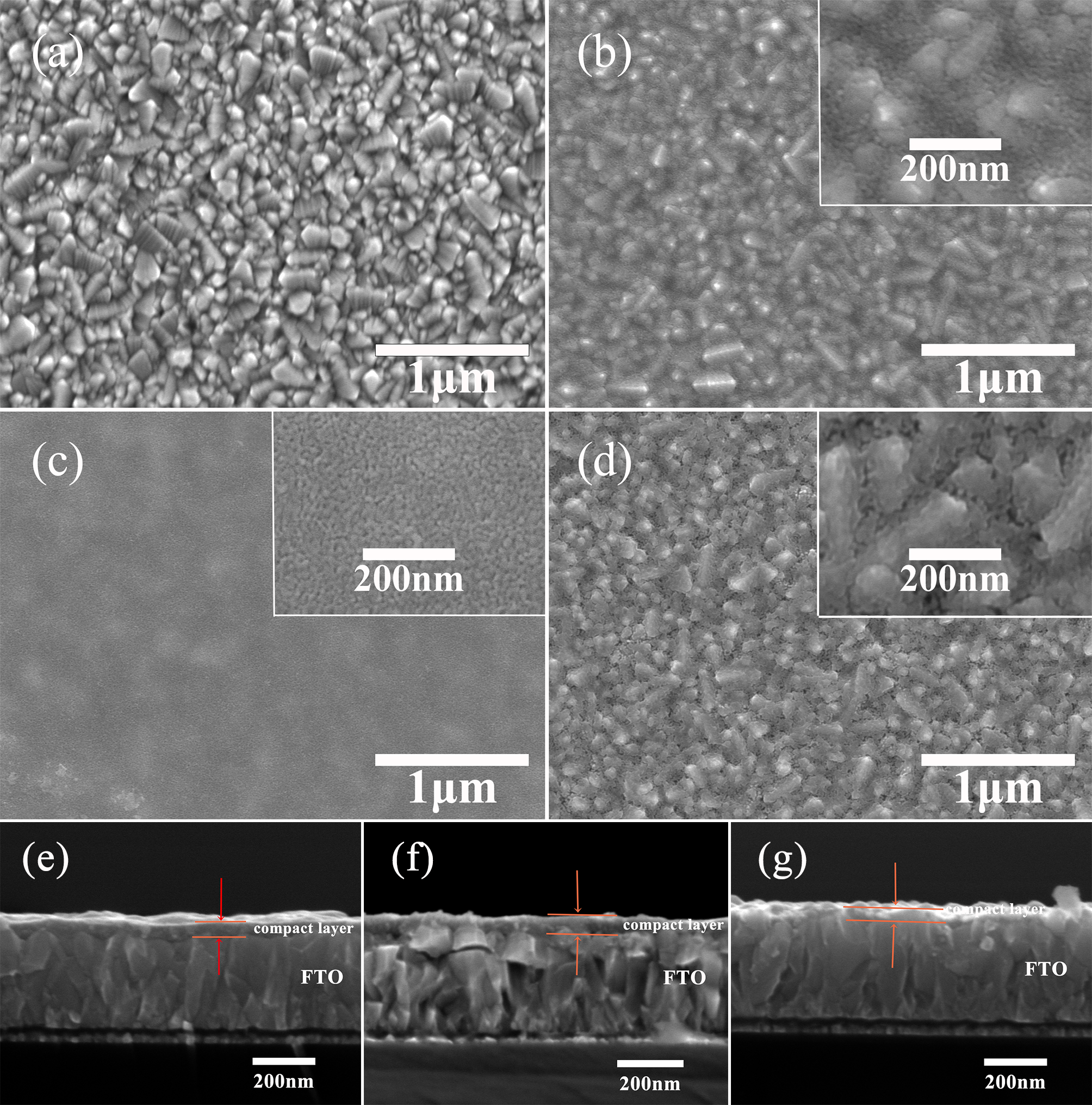


Figure S1 Top-view SEM images of (a) FTO, (b) c-TBOT, (c) c-TTIP and (d) c-TTDB. (Each sample of c-TiO_2_ was recoated for 5 times). Cross-sectional SEM images of (e) C-TBOT, (f) c-TTIP, (g) c-TTDB. (Each sample of c-TiO_2_ was coated for 1 time)


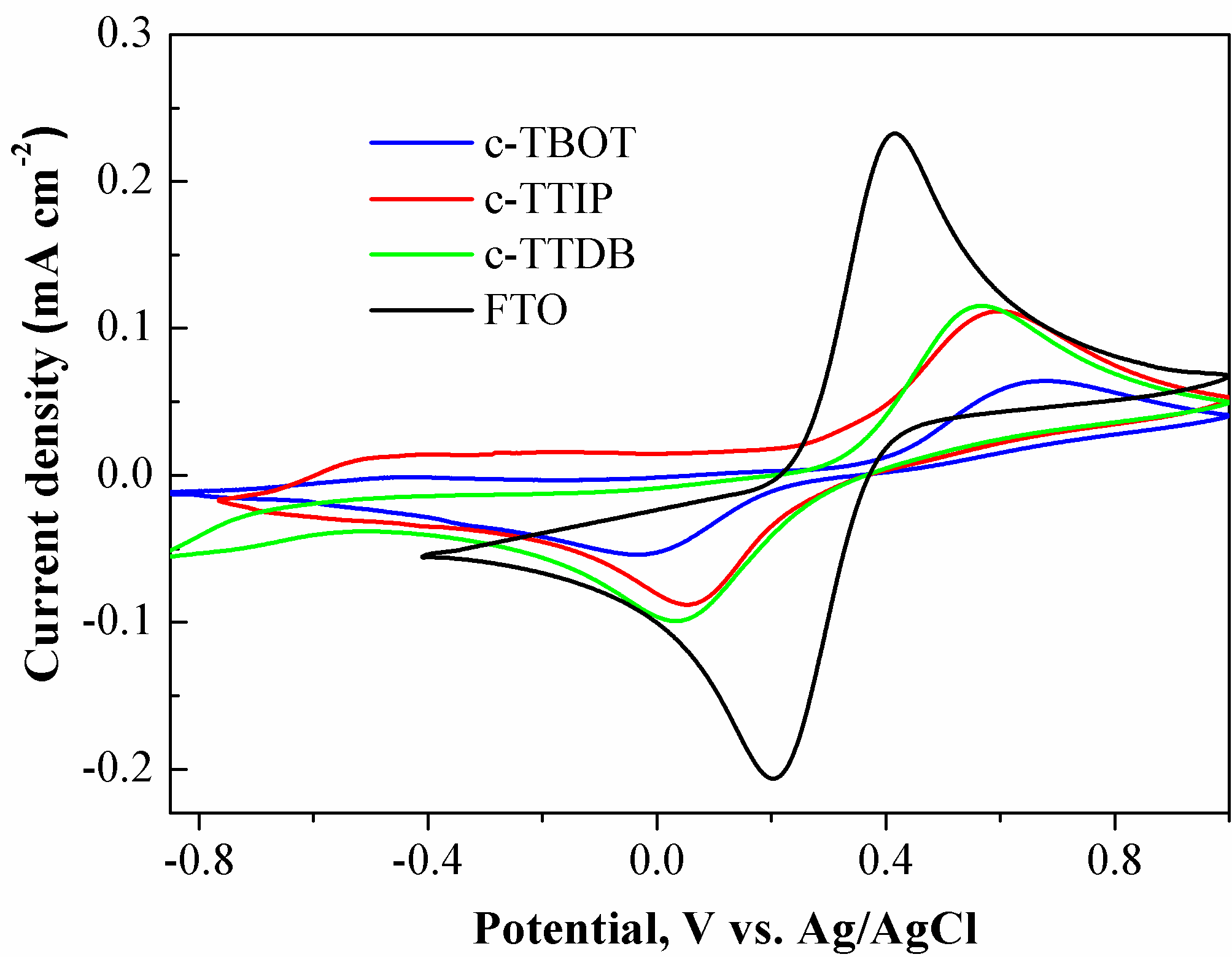


Figure S2 Cyclic voltammograms at bare FTO and that covered with different c-TiO_2_, scan rate: 50mV s^-1^, electrolyte solution: 1mM K_4_Fe(CN)_6_ +1mM K_3_Fe(CN)_6_ in aqueous 0.5M KCl.


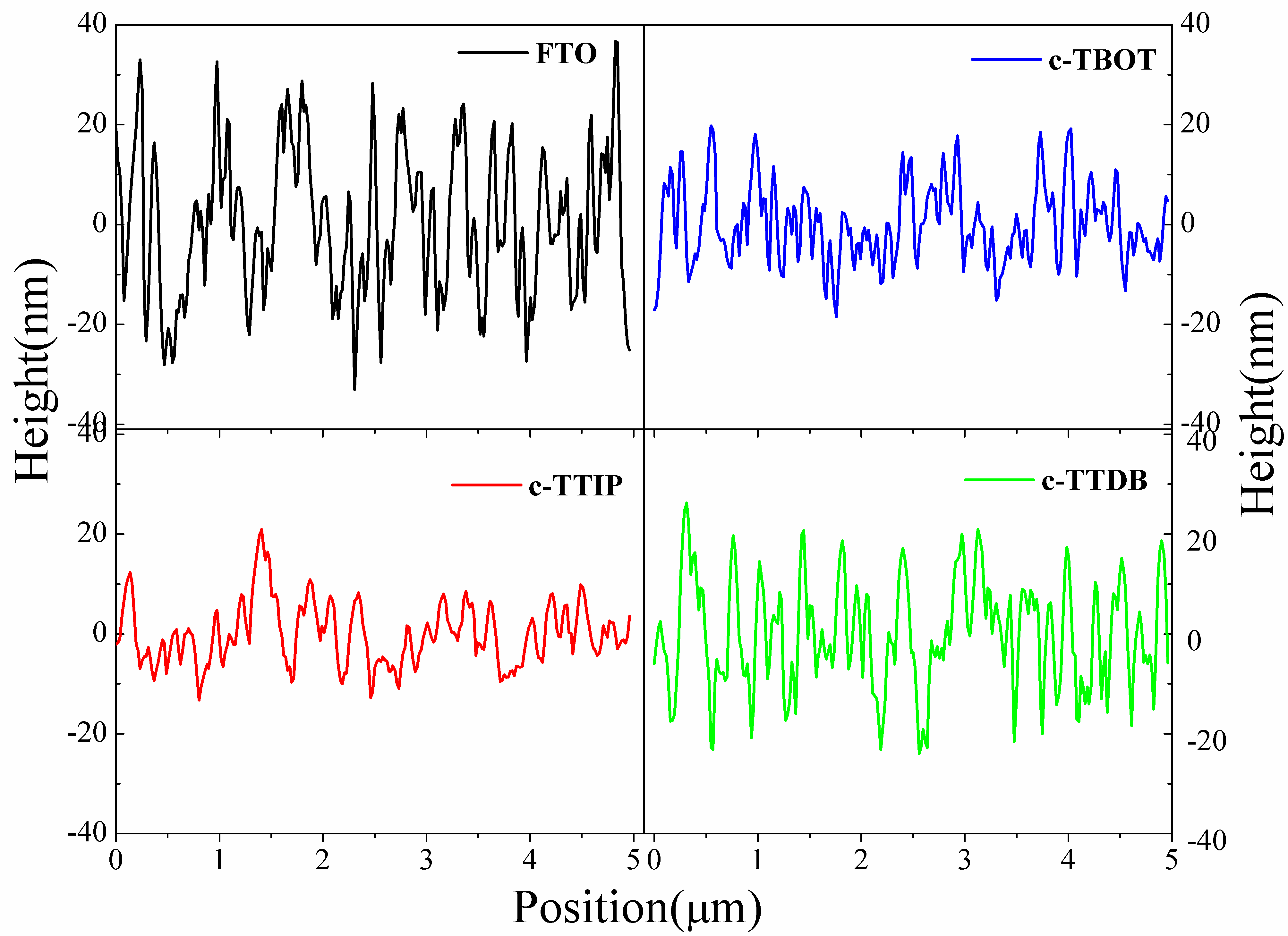


Figure S3 AFM height profiles of the different c-TiO_2_ spin-coating on the FTO glass obtained from the 5μm scale, (a) the underlying FTO, (b) c-TBOT, (c) c-TTIP and (d) c-TTDB. This data can also clearly reveal the roughness of the various substrates

Table S1 Parameters of 5μm × 5 μm AFM roughness

| Compact layer | R_RMS_(nm) | Rα(nm) | Rmax(nm) |
| --- | --- | --- | --- |
| Bare FTO | 13.4 | 10.8 | 85.5 |
| c-TBOT | 9.38 | 7.57 | 73.7 |
| c-TTIP | 6.65 | 5.26 | 61.0 |
| c-TTDB | 11.4 | 9.22 | 77.7 |


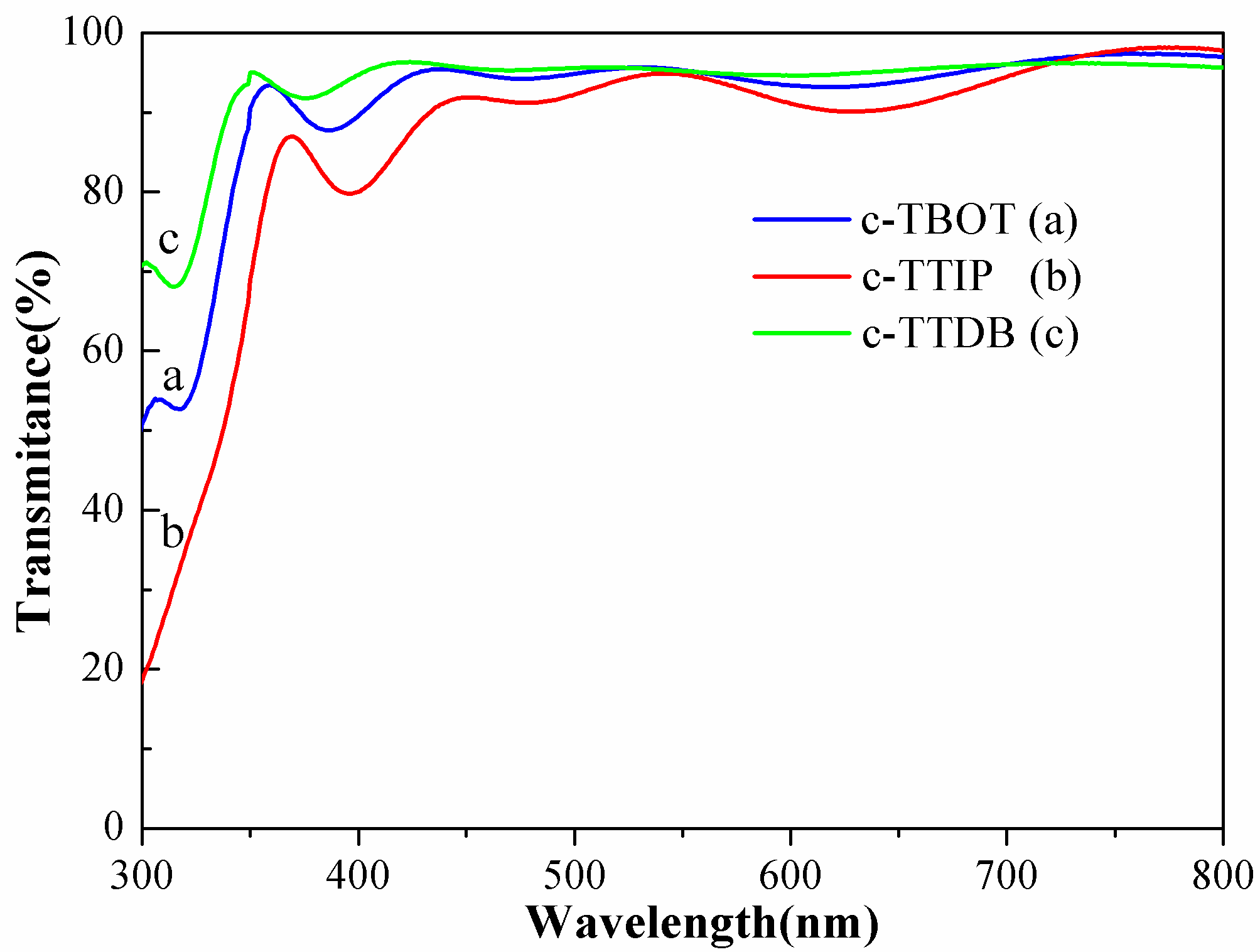


Figure S4 Transmission spectra of different compact films deposited on FTO glass. This data was used to analyze the transmissivity of different c-TiO_2_ in the range between 300 and 800 nm

Table S2 The fitted parameters for EIS equivalent circuit.

| Compact layer | R_s_/Ω cm^2^ | R_rec_/Ω cm^2^ | CPE-T/F |
| --- | --- | --- | --- |
| c-TBOT | 1.907 | 22.040 | 2.003×10^-7^ |
| c-TTIP | 2.198 | 13.680 | 1.719×10^-7^ |
| c-TTDB | 2.201 | 18.750 | 2.407×10^-7^ |
